# A gold nanoparticles-based lateral flow assay utilizing baculovirus expressed recombinant nucleocapsid and receptor binding domain proteins for serodetection of IgG and IgM against SARS-CoV-2

**DOI:** 10.1007/s10529-022-03316-0

**Published:** 2022-11-01

**Authors:** Reda Salem, Amany M. Elshamy, Noha Kamel, Soha Younes, Ohoud M. Marie, Fatma R. Waly, Alaa A. El-Kholy, Wael Elmenofy

**Affiliations:** 1 Agricultural Genetic Engineering Research Institute (AGERI), ARC, Giza, 12619 Egypt; 2 Medical Laboratory Science Department, High Technology Institute of Applied Health Sciences, Badr Academy, Badr City, Cairo, Egypt; 3 Clinical Pathology Department, Faculty of Medicine, Suez Canal University, 4.5 K, Ring Road, Ismailia, 41511 Egypt; 4 Chemistry Department, Faculty of Science, Suez Canal University, Ismailia, 41516 Egypt; 5 Veterinary Sera and Vaccines Research Institute (VSVRI), ARC, Abbassia, P.O. Box # 131, Cairo, 11381 Egypt

**Keywords:** SARS-CoV-2, Lateral flow assay, COVID-19, RBD, Nucleocapsid

## Abstract

Serological assays for SARS-CoV-2 are being utilized at an exponential rate for surveillance programs. This enterprise was designed to develop and validate a qualitative immunochromatographic test, via the Lateral Flow Assay (LFA), for detection of immunoglobulins M and G (IgM and IgG) against both nucleocapsid (N) and the receptor-binding domain (RBD) of the spike protein of SARS-CoV-2. Both targeted proteins were cloned and expressed in baculovirus expression system utilizing insect cells Sf9. The recombinant RBD and N proteins were purified and conjugated with gold nanoparticles (AuNPs) to set up the coating antigens pad. Both anti-human IgG and IgM were dispensed on nitrocellulose membrane to capture human antibodies in serum samples. A home-made dispensing system was developed to draw identical test and control lines. The validity of the developed LFA was verified by testing serum samples from 103 convalescent COVID-19 patients who were PCR positive for SARS-CoV-2 along with 28 control serum samples. The developed strips showed distinctive bands for IgM and IgG of both proteins (RBD and N) in positive samples. The sensitivity of RBD-based LFA was 70.9% and 39.8% for IgG and IgM, respectively, with a specificity of 100% for both. The N-based LFA exhibited a sensitivity of 73.8% and 35.9% for IgG and IgM, respectively, while its specificity was 75% and 100% for IgG and IgM, respectively. Our developed LFA could afford a tool for surveillance programs in low-resource countries. Moreover, it might be functional for rapid and inexpensive monitoring of the anti-SARS-CoV-2 antibodies in the sera of vaccinated individuals.

## Introduction

Severe acute respiratory syndrome coronavirus 2 (SARS-CoV-2) is a highly contagious virus transmitted through human airborne droplets and may trigger systematic and respiratory symptoms although, on some occasions may develop asymptomatic infection (up to 41% of SARS-CoV-2 infections have been caused by asymptomatic cases) (Wang et al. 2020; World Health Organization 2020). Through the fight against the COVID-19 pandemic, the diagnostic performance of different assay approaches for SARS-CoV-2 has been crucial (Marca et al. 2020). Laboratory procedures that shorten the time between testing and results are essential for reducing onward transmission. In addition, PCR technology could be negative after 15 days of infection, and as an antigen-based test, it cannot detect the antibodies which are generated in the circulation against SARS-CoV-2 in the early phase of infection. Moreover, taking swabs require skillfully trained individuals to minimize false-negative results (Crozier et al. 2021).

SARS-CoV-2 genome encodes a number of structural and non-structural proteins. The structural proteins, like spike (S), nucleocapsid (N), membrane (M), and envelope (E), are mainly responsible for genome maintenance, cell membrane attachment, immune evasion, antibodies neutralization, and viral pathogenesis. While, the non-structural proteins, such as RNA-dependent RNA polymerase (RdRp) and protease (3CL), account for translation, transcription, replication, and viral assembly (Fehr and Perlman 2015).

Meanwhile, spike protein has the fundamental role in the stimulation of immune responses and initiation of antibody neutralization process. It has the most divergent mutations in the non-conserved regions which contribute to the antigenic variations in various SARS-CoV-2 genotypes. Regarding the role of protein N in viral infection, it is the most abundant and highly immunogenic expressed protein that has the ability to elicit the antibodies response (Ahmed et al. 2020; Ying et al. 2003). The rapid growth of SARS-CoV-2 infections worldwide with a variety of clinical signs has been attributed to the emergence of mutations with several related variants. These mutations were reported mostly at loci on the receptor-binding domain (RBD) of spike gene and in part on the N gene (Ziegler et al. 2020). Therefore, studying those proteins is the central target for each scientist to assist control of the current COVID-19 pandemic.

Serological approaches such as Enzyme-linked immunosorbent assay (ELISA) and Lateral Flow Assay (LFA) have a fundamental role in the detection of antibodies against pathogens. Serological approaches, such as LFA, have many limitations with lower sensitivity compared to PCR technology. However, they are based on serum sample, which is less painful to apply and does not need skillful operators (Li et al. 2021). It has been reported that some emerging SARS-CoV-2 mutations had a variable impact on the performance of the molecular diagnostic assays depending on nucleotide mismatch and position on the targeted genomic sequence (Ziegler et al. 2020). Hence, subsequent efforts were to be conducted to verify how far the rising SARS-CoV-2 mutations would impact the performance of such assays and to ensure their diagnostic utility.

Seroconversion to SARS-CoV-2 is manifested by the onset of specific antibodies IgM (~ 3–6 days) and IgG (~ 14 days) post-infection (Guo et al. 2020). Additionally, a study has demonstrated that seroconversion of IgM and IgG may be detectable after a few days (2–23 days) as clinical symptoms start (Long et al. 2020).

In this endeavor, we intended to develop and validate a colloidal gold-based immuno-chromatographic strip using SARS-CoV-2 recombinant N and RBD proteins, cloned and expressed in the baculovirus-insect cells expression system. Moreover, the developed LFA was verified for its utility in serodiagnosis of SARS-CoV-2 infected patients by specific detection of early IgM and late IgG antibodies in serum samples.

## Materials and methods

### Serum samples

A total of 103 convalescent Egyptian cases with recorded positive RT-PCR for SARS-CoV-2 infection, from a private accredited Medical Laboratory, were enrolled in this retrospective study. All serum samples were collected after written informed consent from the patients, from November 2020 to January 2021. Patients’ clinical history was collected in accordance with the Declaration of Helsinki and was approved by the Research Ethics Committee, Faculty of Medicine, Suez Canal University with reference number #4597.

Of the 103 cases, female (n = 45; 43.6%) and male (n = 58; 56.3%) patients, 16 cases suffered from severe illness (SpO_2_ < 94% on room air, a respiratory rate > 30 breaths/min, and lung infiltrates > 50%); whereas, the other 87 cases went through mild to moderate symptoms of COVID-19. All sera were collected at 14-days after the onset of clinical symptoms (up to 50 days in severe cases).

As a control group, 28 sera have been collected from healthy female (n = 11) and male (n = 17) cases prior to the pandemic, from November 2018 till June 2019, were used. Samples from both SARS-CoV-2 convalescent and healthy cases were collected following firm exclusion criteria were applied: ages of ˂10 years and > 80 years; positive cases for Hepatitis C/B and HIV, along with immunocompromised patients.

All samples were heat-inactivated at 56 °C for 30 min and kept at -20 °C to be tested. Handling of samples and assays were performed in a Biological Safety Level 3, as needed, according to the guidance of the Center for Disease Control and Prevention (Sick 2020).

### Generation of recombinant SARS-CoV-2N and RBD antigens

#### Designing, synthesis, and cloning of the N and RBD genes

The open reading frames encoding for the N and RBD proteins of SARS-CoV-2, derived from the nucleotide sequence of the reference isolate Wuhan-Hu-1 (GenBank accession number: NC_045512.2), were cloned, expressed, and purified according to our lab’s established and modified protocols (Salem et al. 2019a, 2020, 2022).

The corresponding nucleotide sequences (N and RBD) were subjected to codon usage optimization to better matched expression profile of *Spodoptera frugiperda* (the surrogate cells used for expression). The coding sequence of 6-Histidine residues and an enterokinase recognition sequence were inserted at the 3´ end of N and RBD coding sequences. The synthesized sequences were cloned into the BssHII/PstI sites of the pFastBac cloning vector and expression was derived by the Ppol promoter (Salem et al. 2019b; Elmenofy et al. 2020; Sheikhzadeh et al. 2020).

#### Generation of recombinant baculoviruses

To generate recombinant baculoviruses harboring the N and RBD genes, the hybrid pFastBac1-N and pFastBac1-RBD plasmids were transformed into DH10Bac *E. coli* cells following the manufacturer's instructions (Thermo-Fisher). In brief, the constructed hybrid plasmids were individually transformed into DH10Bac cells. This allows the cassettes carrying the N or RBD genes between Tn7R and Tn7L sequences in pFastBac1 to be transferred into the bacmid in DH10Bac cells through site-specific transposition. Recombinant baculoviruses were then isolated, screened, amplified, and titrated according to the standard methods provided with the Bac-to-Bac baculovirus system (Thermo-Fisher). The N and RBD recombinant proteins were produced via infection of Sf9 cells, with the generated recombinant baculoviruses. The insect cell line Sf9 (Thermo-Fisher) was grown and maintained at 27 °C using ExCell-420 Serum-Free medium (Sigma-Aldrich), supplemented with 10 µg Gentamycin. It was used in all procedures of baculovirus expression of SARS-CoV-2 N and RBD genes, preparation of recombinant baculovirus stocks and production of the recombinant proteins (Scholz and Suppmann 2017).

Infected cells were cultured in T-flasks for small-scale production, and culture supernatants, as well as infected cells, were harvested daily at 3–7 days post-infection (DPI). Each harvest was subjected to sodium dodecyl sulphate–polyacrylamide gel electrophoresis (SDS-PAGE) and western blot analyses (El-Gaied et al. 2017; Salem et al. 2021), to verify integrity and immunoreactivity of the expressed proteins, as well as their peak-expression times.

#### Purification of N and RBD proteins

Five days post-infection, infected Sf9 cells using the recombinant baculoviruses were collected and total protein was estimated. The culture supernatants were tested for secretory N and RBD recombinant proteins by SDS-PAGE. The N and RBD polypeptides fused with N-terminal 6xHis-tag were purified from the re-suspended cell pellets using Ni–NTA agarose resin (Qiagen, Germany). Finally, the recombinant N and RBD containing imidazole were dialyzed in 1 × phosphate-buffered saline (PBS) (pH 7.4) for overnight at 4 °C, and the concentrations were determined using the Bradford protocol (Kielkopf and Bauer 2020).

### Mice immunization

Two female BALB/c mice, 21 days old were obtained from Theodor Bilharz Research Institute, Giza, Egypt, and treated in compliance with the principles and policies of the American Physiological Society's Guiding Principles in the Care and Use of Animals and after the approval of research ethics committee, Faculty of Medicine, Suez Canal University. Mice were injected with the recombinant N and RBD proteins; according to the mentioned injection schedule. The primary immune response was initiated by intraperitoneal injection of mice with 50 μg of recombinant protein (N or RBD) emulsified in complete Freund’s adjuvant, followed by 4 (each/week) subsequent intravenous boosters, each with 100 μg protein emulsified in incomplete Freund’s adjuvant that was excluded from the last booster. Seven days post the 5th injection, sera were collected for assessment of anti- N and RBD seroconversion; then mice were euthanized and spleens were gathered for isolation.

### Preparation of colloid gold nanoparticle

Gold nanoparticles (AuNPs) with a diameter of about 30 nm were prepared by citrate-based reduction method according to Turkevich Method (Dong et al. 2020). Briefly, 1 mL of 1% sodium citrate was added to a 99 mL of 0.01% stirred boiling HAuCl4 solution. After vigorous stirring with continuous boiling, till the solution’s color has turned from purple to red wine (approximately for 3–5 min). With no longer changes in color, the solution was allowed to gradually cool down at room temperature. The colloid AuNPs concentration was checked by Spectrophotometer at a wavelength of 525 nm (Beckman DU 530 UV–Vis, USA) and their particle size was confirmed by transmission electron microscopy (TEM, Jeol JEM-1400, Japan), as has been described (Zhang et al. 2009).

### Preparation and optimization of protein-gold conjugate

For preparing the protein-AuNPs conjugate, serial pH values of gold solution were prepared by 0.2 M potassium carbonate solution. In addition, several protein concentrations were prepared and added to the colloidal gold solutions (pH adjusted) (Zhang et al. 2009). After 30 min of incubation, a 125 µl of 1 M sodium chloride (NaCl) was added to the reaction to induce the aggregation effect on the gold particles. The optimal amount of protein required for gold stabilization was determined by spectrophotometer at 530–650 nm (Zhang et al. 2009; Busch et al. 2019).

To block the surface sites on AuNPs, increasing the colloidal AuNPs stability and avoiding unspecific interactions, the optimized concentrations of dialyzed recombinant proteins N and RBD were separately added to 1 ml of adjusted pH gold solution and incubated for 1 h with shaking, then, a 125 µl of 10% bovine serum albumin (BSA) was added. After extra 15 min of incubation with stirring, the prepared AuNPs were washed with 250 µl of BSA (1%) for three times then, centrifuged for 30 min at 15,000 rpm and 4 °C. Eventually, the supernatants were discarded and the pellets (containing antigen-gold conjugate) were distinctly re-suspended in a 100 µl of conjugation buffer (0.5% BSA, 50 mM Tris–HCL, 0.5% Tween 20, 2% sucrose) and kept at 4 °C (Zhang et al. 2009).

### Assembly and evaluation of the Lateral Flow strips

**Fig. 1:**
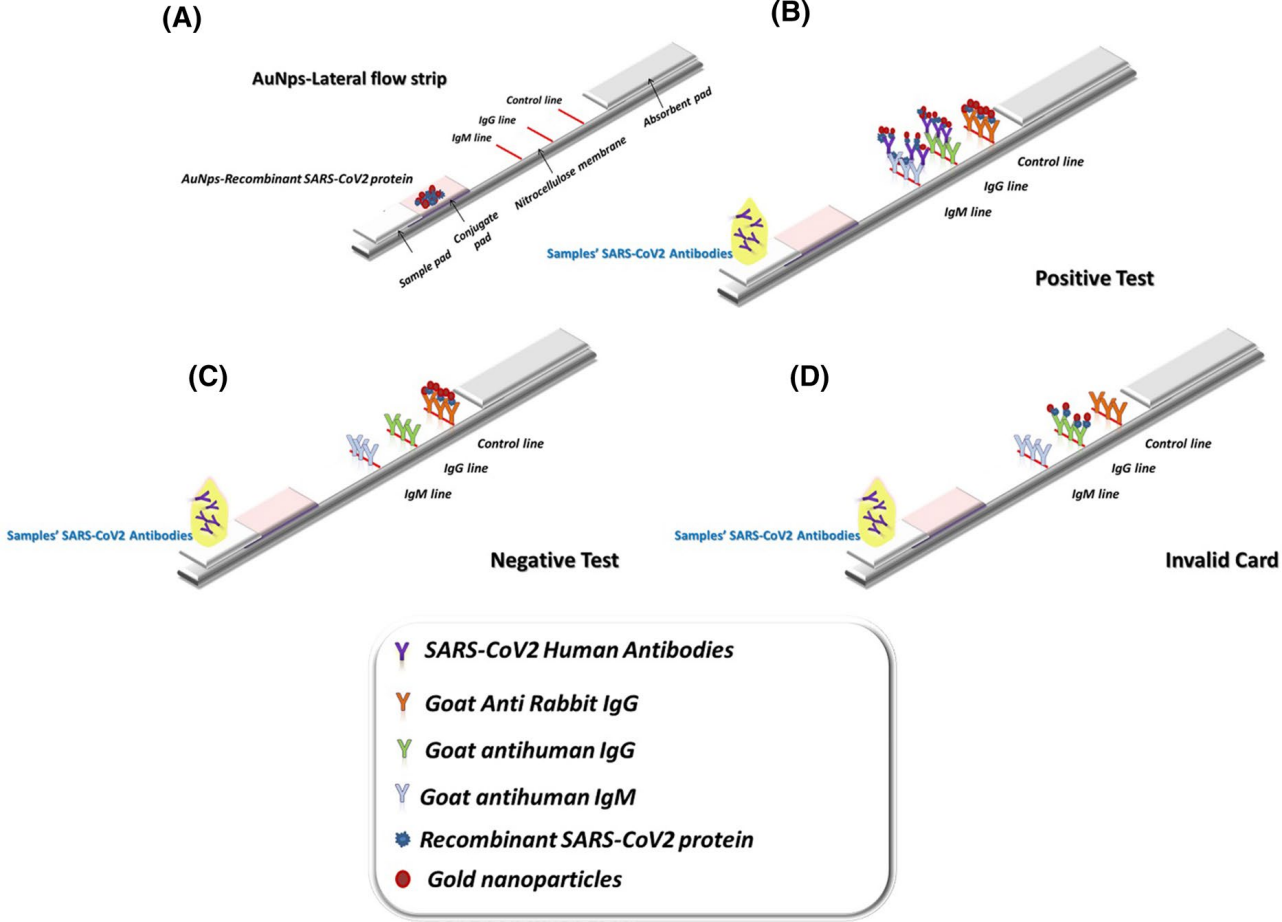
The basic structure of the developed AuNPs-Lateral Flow Assay. **A** Principle structure of AuNPs-Lateral Flow strip. **B** Positive reaction of the developed strip. **C** Negative reaction. **D** Invalid reaction

The structural design of the developed strips is demonstrated in (Fig. 1). The components of the Lateral Flow strip were assembled into an adhesive-backing card including: nitrocellulose membrane (3 × 10 cm), absorbent pad (2.5 × 10 cm), conjugate pad (1.5 × 10 cm), sample pad (1.5 × 10 cm) and adhesive double taps. Briefly, the conjugate pad was dipped in the conjugate solution containing 20 mM sodium borate (pH 8.5), BSA (2%), sucrose (3%), NaCl (0.6 M), Tween 20 (0.2%), and sodium azide (0.1%). It was then left for 30 min at 50 °C to dry. To prepare the sample pad, it was treated with PBS (pH 7.2) containing NaCl (0.1 M), Tween 20 (0.2%), and sodium azide (0.1%), then left to dry for 30 min at 50 °C (Zhang et al. 2009, 2006). As test lines, Goat anti-human IgG (Cat.#: 31,130, 2.4 mg/mL), and Goat anti-human IgM (Cat.#: A18843, 2.5 mg/mL) were dispensed onto the nitrocellulose membrane, whereas, the control lines were contained purified mice IgGs against recombinant N and RBD to SARS-CoV-2. The space between each line was ~ 0.5 cm. The blotted membrane was blocked with BSA (1%) and allowed to dry at 37 °C for 2 h.

For assembly, firstly; the blotted membrane was stamped in the middle followed by an adsorption pad with 0.5 cm over-crossing at the adsorption pad end. Both sample and conjugate pads were affixed with 0.5 cm overlapping at the end of the conjugate pad. Then, pads were fixed next to the blotted membrane with 0.5 cm overlapping at the end of the conjugate pad. The strips were then sealed and stored at 4 °C till used.

For evaluation, fifty µl of infected serum sample was directly loaded into the sample pad on the strip, followed by 50 µl of PBS. Detection was occurred by providing a strong and reliable band on test and control lines. Lateral flow for IgM and IgG sensitivity and specificity as 95% confidence intervals were estimated, and Cohen’s Kappa was calculated (Landis and Koch 1977).

## Results and discussion

### Production and purification of N and RBD proteins

**Fig. 2:**
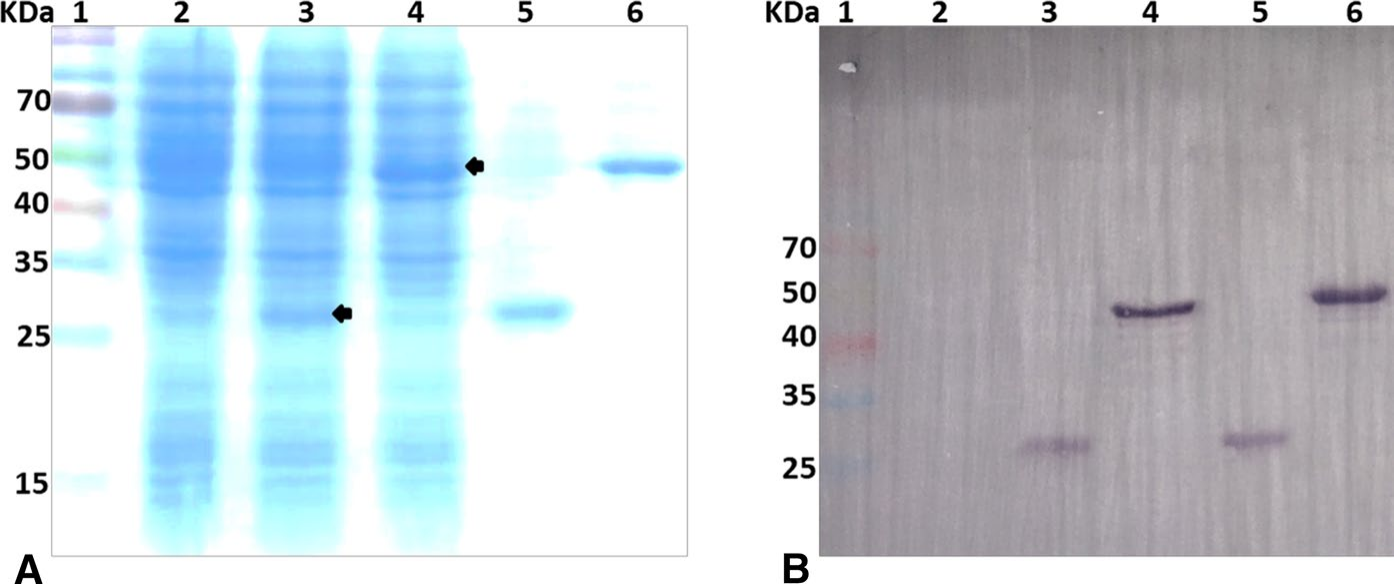
**A** SDS-PAGE showing the expression and purification of recombinant RBD and N proteins. 1: protein ladder; 2: negative control (total protein lysates from non-infected Sf9 cells); 3 and 4: showing the expression of RBD and N, respectively in the Sf9 cell lysates infected with the corresponding recombinant baculovirus; 5 and 6: purified RBD and N, respectively, purified using Ni–NTA affinity chromatography. **B** Confirming the identities of the SDS-PAGE polypeptides by western blotting

The successful generation of recombinant baculoviruses, harboring the proper nucleotide sequences encoding for the N and RBD cloned genes of SARS-CoV-2, was verified by specific PCR (data not shown). Expression of both recombinant proteins (N and RBD) in Sf9 insect cells infected with the recombinant baculoviruses were demonstrated via SDS-PAGE and western blot. As shown by SDS-PAGE (Fig. 2A), discrete protein bands at the size of ~ 46 KDa and ~ 21 KDa were evident for the expressed N and RBD recombinant proteins, respectively, in the Sf9 cell lysates infected with the corresponding recombinant baculovirus, where were absent in lysates from non-infected Sf9 cells. Furthermore, the recombinant N and RBD proteins fused to His-tag were purified using Ni–NTA affinity chromatography. The identities and immunoreactivity of these polypeptides were also confirmed by western blotting (Fig. 2B).

### Characterization of gold nanoparticles

**Fig. 3:**
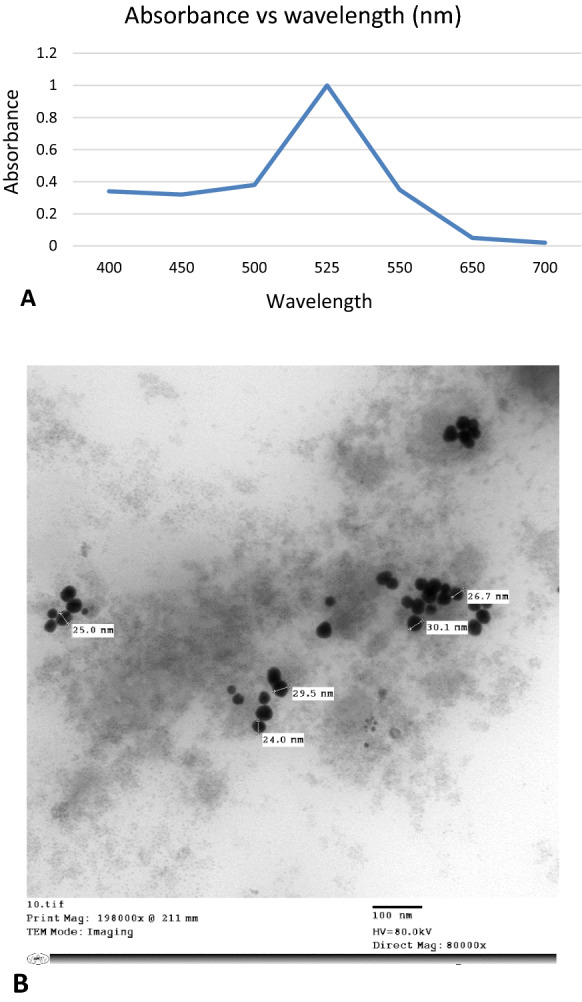
**A** Optimal size for the prepared gold nanoparticles (AuNPs) as the maximum optical density (OD = 1) noticed at the wavelength of 525 nm. **B** TEM imaging showing the uniformity, homogeny, morphology, and distribution of the prepared AuNPs

Uniformity, homogeny, morphology, and distribution of the prepared AuNPs were characterized by Spectrophotometry and TEM imaging. Results shown in (Fig. 3A) indicated obtaining the optimal size of prepared AuNPs as the maximum optical density (OD = 1) noticed at the wavelength of 525 nm (Zhang et al. 2009). Regarding TEM images, the gold particles were spherical with uniformity and sound-dispersion in a good approximation. The average size of particles was approximately 25–30 nm as shown in (Fig. 3B).

### Conjugation of prepared AuNPs with recombinant proteins

The conjugation of AuNPs with recombinant proteins was conducted by the physical electrostatic interaction. Basically, in this type of conjugation, the pH value is a critical point and should be in a range of ± 0.5 around the protein's isoelectric point. Thus, a range of pH values (6.0, 6.5, 7.0, 7.5, 8.0, 8.5, 9.0, 9.5, and 10) were set. We found that the optimal pH value for conjugation was 9 (as the wine-red color was formed). Conversely, the other pH values exhibited a purple or white–gray color, which indicating the aggregation and destructive effect for gold particles.

Furthermore, to add the optimal protein’s amount for conjugation, different concentrations of the recombinant proteins 1, 3, 5, 10, 20, 30, 40, 50, 60, 70, 80, 90, 100, and 110 µg/ml were prepared using the adjusted pH gold solution (pH 9). We found that a 70 to 80 µg/ml and 60–70 µg/ml of recombinant N and RBD, respectively were the optimal concentrations for conjugation.

### Optimization of the lateral flow strips

**Fig. 4:**
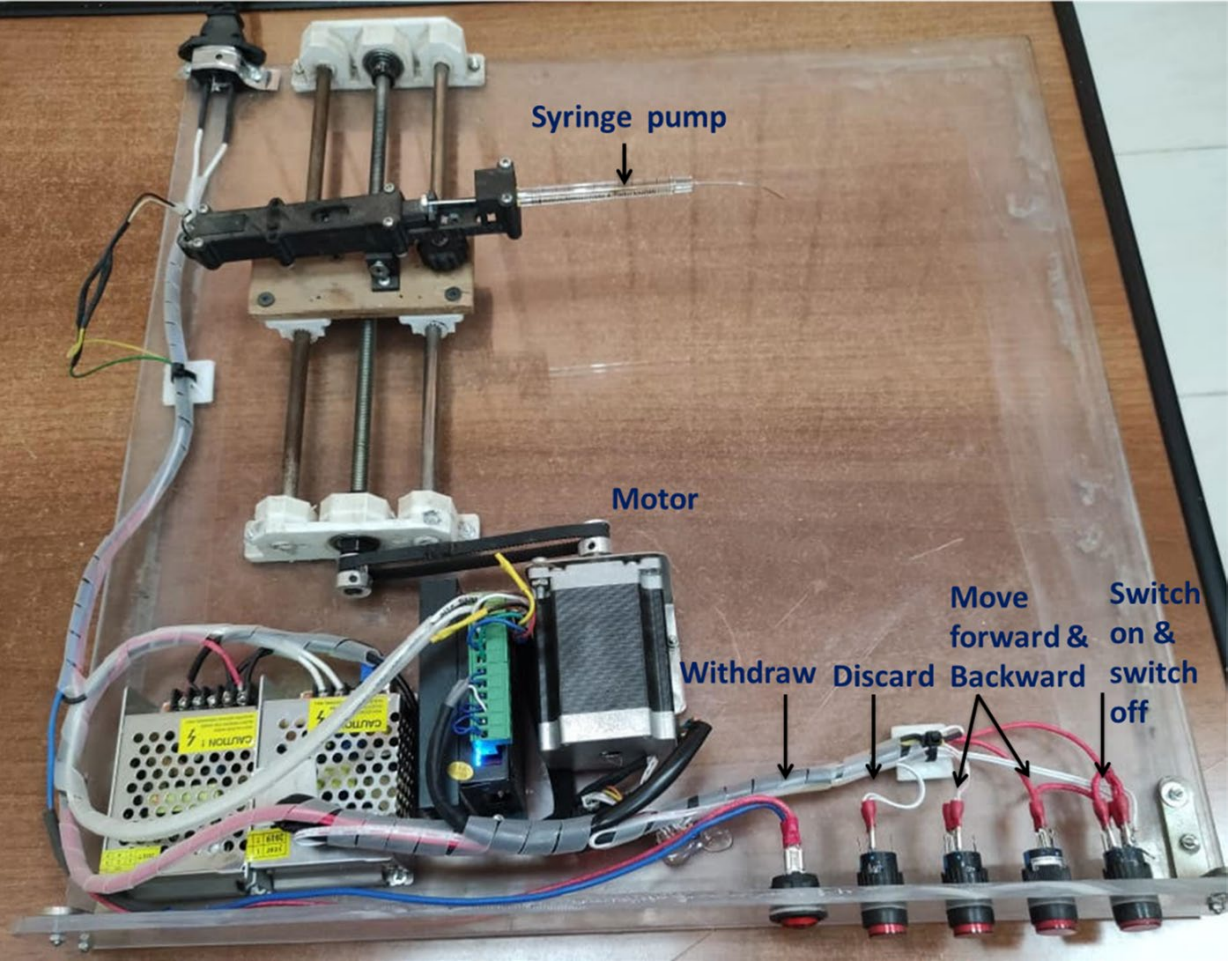
Home-made developed dispensing system

For accurate dispensing of the used antigens and antibodies on the lateral flow membranes, we developed a home-made system (Fig. 4) to make precise lines (test and control). For control lines, the mice purified IgG (raised against recombinant proteins) was tested in different concentrations 0.5, 1, 1.5, 1.8, and 2 mg/ml. Results showed that the optimal IgG concentration 2 mg/ml and 1.8 mg/ml for N and RBD strip, respectively. Regarding the test lines, the most favorable concentrations were 1.0 and 1.2 mg/ml for Anti- IgG and IgM, respectively.

To demonstrate clear control and test lines, avoiding non-specific binding, and eliminating the false-positive results, 1% of bovine serum albumin (BSA) was used to block the nitrocellulose membrane and this is consistent with previous studies (Huang et al. 2020; Wen et al. 2020). Notably, the strips that were blocked with 2% or 0.5% or 0.0% of BSA exhibited unreliable bands and a deepened red background with false-positive bands in the control sera.

In addition, for sampling, various dilutions of serum samples 1:1, 1:2, 1:3, 1:4, 1:5, 1:6, and 1:8 in PBS (pH 7.2) were prepared. The test bands revealed shortcomings except the dilution 1:4 which demonstrated a slightly clear result which is in a contrary with Huang et al. 2020. However, direct loading of sample (50 µl) onto the strip, followed by a 50 µl of PBS resulted in strong and trustworthy bands to distinguish. Notably, less than 20 µl of the direct sample loading provided negligible bands and thus leading to unreliable results.

### Sensitivity and specificity of the lateral flow strips

To evaluate the developed Lateral Flow strips, human serum samples of 103 convalescent COVID-19 patients (previously proven by PCR) were tested. These patients were of median age of 41.5 years; a group with severe illness (n = 16) and a group with mild-to-moderate illness (n = 87), as (IQR: 22-62ys), 46 years (IQR: 22-65ys); respectively. For the severe illness group, the median period was 32 days from symptoms onset to samples withdrawal, while the median period for the mild-to-moderate symptoms group was 23 days. In addition, 28 serum samples that had been collected prior to the pandemic were used as negative controls.

Each sample was tested in duplicate and results were observed by the naked eye. Data in (Table 1) provided the sensitivity and specificity of LFA that developed based on IgG against N and RBD proteins calculated upon the PCR results. RBD-based LFA IgG sensitivity was 70.9% and specificity was 100%. While, N-based LFA IgG sensitivity was 73.8% and 75% specificity. A value of 0.51 of Cohen’s Kappa was obtained revealing a moderate agreement between RBD-based LFA IgG and PCR; while, a fair agreement with a value of 0.39 was obtained for N-based LFA IgG.

**Table 1:** RBD and N IgG lateral flow assay (LFA) test compared to positive SARS-CoV-2 reverse transcription polymerase chain reaction (RT-PCR) from nasopharyngeal swab (103 patients and 28 control)

Specificity	Sensitivity	PCR		Result	
		Neg	Pos		
100%	70.9% (95% CI)	0	73	Pos	RBD-based LFA IgG
		28	30	Neg	
75%	73.8% (95% CI)	7	76	Pos	N-based LFA IgG
		21	27	Neg	

While, Table 2 provided the sensitivity and specificity of LFA based on IgM against N and RBD proteins calculated upon the PCR results. PCR and RBD-based LFA IgM sensitivity was 39.8%, while specificity was 100%. N-based LFA IgG sensitivity was 35.9% with 100% specificity and a value of 0.22 of Cohen’s Kappa for RBD-based LFA IgG, which is a fair agreement. While a slight agreement with a value of 0.19 was obtained for N-based LFA IgG.

**Table 2:** RBD and N IgM lateral flow assay (LFA) test compared to positive SARS-CoV-2 reverse transcription polymerase chain reaction (RT-PCR) from nasopharyngeal swab (103 patients and 28 control)

Specificity	Sensitivity	PCR		Result	
		Neg	Pos		
100%	39.8% (95% CI)	0	41	Pos	RBD-based LFA IgM
		28	62	Neg	
100%	35.9% (95% CI)	0	37	Pos	N-based LFA IgM
		28	66	Neg	

Table 2 RBD and N IgM lateral flow assay (LFA) test compared to positive SARS-CoV-2 reverse transcription polymerase chain reaction (RT-PCR) from nasopharyngeal swab (103 patients and 28 control).

Both molecular and serological methods could give false results because of incorrect sampling, insufficient viral material, improper RNA extraction, non-specific cross-reactivity with other viruses, contamination or technical issues. Although the serological tool could enhance identification capacity, particularly during pandemics, and they provide rapid and affordable screening programs to control or mitigate the infection. The relatively low sensitivity and specificity is a major limitation. Possible improvement strategy focusing on identifying novel signal augmentation approaches and quantification systems warrant further study. So, our next step is to develop an ELISA with a quantitative or semi-quantitative approach in which enzymatic reactions could be used to improve the sensitivities and specificities.

Here, we developed the first Egyptian Lateral Flow platform to evaluate the IgG and IgM antibodies against SARS-CoV-2 infection in human serum. PCR-based technology was performed on virus-containing specimens with extreme risk, requiring exceptional biosafety precautions, intensive labor with well-trained skills to mitigate the invalid results. Furthermore, its limited and costive testing capacity, the exposure time to the virus, the onset of symptoms and the optimal time of viral detection are critical elements to detect the infection by PCR. Alternatively, the point of care test such as LFA is an easy-to-handle tool, could boost the identification capacity and provide the result within 20 min. The developed strips provide sensitivity 70.9% and 73.8% for RBD-based IgG and N-based IgG, respectively. In a previous study, the gold nanoparticle-Lateral Flow strip (AuNPs-LF) was developed with a sensitivity value of 69.1% (Wen et al. 2020). Also, Zeng et al. (Zeng et al. 2020) demonstrated that the positive rate of the single IgG-RBD was 61.76% for the AuNPs-LF strip. In the current study, the level of agreement between developed RBD-based LFA IgG and the PCR tool was 0.51 (Cohen’s Kappa), compared to 0.39 for N-based LFA IgG. This might be due to the highly conserved N-terminal domain of the N protein of beta-coronaviruses compared to RBD-protein which is only specific to SARS-CoV-2.

Interestingly, the IgM antibodies arise gradually during the first week of infection and may decline rapidly within two weeks. Unlike IgM, the IgG is elicited after 10–14 days of exposure and persist in sera (over 48 days) for an extended period of time (Guo et al. 2020; Hou et al. 2020).

In this study, the median time for detection of the SARS-CoV-2 antibodies in serum samples was 32 days from the onset of symptoms. This might explain why the patients in this study were seropositive for IgG more than IgM. Besides, this would clarify why sensitivity for the RBD-IgM and N-IgM, in this study, was 39.8% and 35.9%, respectively. Consequently, IgM serological assay is potentially accounting for poor sensitivity after one month of infection. Thereby, it was recommended to combine the IgG and IgM in a diagnostic test designed to detect SARS-CoV-2 infection rather than using a single antibody testing (Li et al. 2020).

## Conclusion

During the pandemic, the need for a test that is easy, rapid, portable and affordable, was raised for screening and controlling the spread of infection. LFA is a promising preliminary serological test. Although, it is fast, affordable, and easy to use, it should only be used for serological surveillance. A clinical correlation and confirmatory test should be the basis for starting treatment. The generated LFA will be used in a future study for monitoring the antibodies against SARS-CoV-2 after vaccination and in quality control assessment for developed vaccines. The developed assay initiate a potential for future upgrading of COVID-19 routine diagnostic agenda as it could be deployed in low-economy countries or regions at risk. Rapid, accurate, simple and inexpensive diagnostic tests are advantageous as they provide better patient triage and treatment, lessening the spread of outbreaks by fast recognition of infected persons, appropriate to lab facilities at regular clinics without the need for a skilled practitioner, and lower testing costs. The developed LFA utilizes a complete single-use, room thermo-stable test cartridge, which is appropriate for early (IgM) and late (IgG) rapid serodiagnosis of suspected SARS-CoV-2 infected patients, and might be a useful tool for quality control of vaccines and sero-monitoring of vaccinates as well.

## Data Availability

The datasets used and/or analyzed during the current study are available from the corresponding author on reasonable request.
